# LAMPS: an analysis pipeline for sequence-specific ligation-mediated amplification reads

**DOI:** 10.1186/s13104-020-05106-1

**Published:** 2020-06-03

**Authors:** Christopher J. F. Cameron, Xue Q. D. Wang, Josée Dostie, Mathieu Blanchette

**Affiliations:** 1 School of Computer Science, McGill University, Montréal, Canada; 2 Department of Biochemistry and Goodman Cancer Research Center, McGill University, Montréal, Canada

**Keywords:** Bioinformatics pipeline, Multiplexed ligation-mediated amplification, Carbon copy-chromatin immunoprecipitation, Chromosome Conformation Capture Carbon Copy

## Abstract

**Objective:**

Ligation-Mediated Amplification (LMA) is a versatile biochemical tool for amplifying selected DNA sequences. LMA has increased in popularity due to its integration within chromosome conformation capture (5C) and chromatin immunoprecipitation (2C-ChIP) methodologies. The output of either 5C or 2C-ChIP protocols is a single-read sequencing library of ligated primer pairs that may or may not be multiplexed. While many computational tools currently exist for read mapping and analysis, these tools neither fully support multiplexed libraries nor provide qualitative reporting on the LMA primers involved. Typically, the task of library demultiplexing or primer analysis is offloaded on to the user. Our aim was to develop an easy-to-use pipeline for processing (multiplexed) single-read sequencing data produced by sequence-specific LMA.

**Results:**

Here, we describe the Ligation-mediated Amplified, Multiplexed Primer-pair Sequence (LAMPS) analysis pipeline. LAMPS facilitates the analysis of multiplexed LMA sequencing data and provides a thorough assessment of a library’s reads for a variety of experimental parameters (e.g., primer-pair efficiency). The standardized output of LAMPS allows for easy integration with downstream analyses, such as data track visualization on a genome browser. LAMPS is made publicly available on GitHub: https://github.com/BlanchetteLab/LAMPS

## Introduction

Ligation-Mediated Amplification (LMA) library preparation protocols [1, 2] have become increasingly useful in targeted sequencing methodologies. Applications include Carbon Copy-Chromatin Immunoprecipitation (2C-ChIP) [3], used to study protein-DeoxyriboNucleic Acid (DNA) interactions at a defined set of loci, and Chromosome Conformation Capture Carbon Copy (5C) [4], for the targeted analysis of chromatin architecture. These assays generally produce single-read sequencing data and are often highly multiplexed. The specificity of the protocol makes it difficult to use generic bioinformatics pipelines for sequencing data analysis, such as Burrows-Wheeler Aligner’s Smith-Waterman (BWA-SW) [5], Quantitative Insights Into Microbial Ecology (QIIME) 2 [6], or Torrent Mapping Alignment Program (TMAP) [7] (see Table 1). For example, the ligated, sequence-specific Forward (F) and Reverse (R) primers used in LMA may be incorrectly labeled as Polymerase Chain Reaction (PCR) duplicates or sequencing artifacts. Due to the low error rate of high-throughput sequencing and length of ligated primer-pair sequences, LMA reads are very similar and will map to the same genomic loci. In addition, analyses of LMA primer-pair (amplification) efficiencies for target DNA sequences are not standard. Users may also need to separately pre-process the sequencing data to demultiplex it. There exists a need for a computational pipeline that can more easily process LMA sequencing data, while providing diagnostic feedback on a variety of common issues that arise in this type of applications (i.e., primer-pair efficiency).

**Table 1:** Comparison of single-read mapping software

	BWA-SW [5]	QIIME 2 [6]	TMAP [7]	LAMPS
Handles multiplexed reads	✗	✓^a^	✗	✓
Primer QC reports provided	✗	✗	✗	✓
2C-ChIP library normalization	✗	✗	✗	✓
Genome browser formatted output	✗	✗	✗	✓
Software dependencies	C Javascript	Python	C Python	Python Bowtie 2

^a^Using the ‘q2-cutadapt’ plugin

**Fig. 1:**
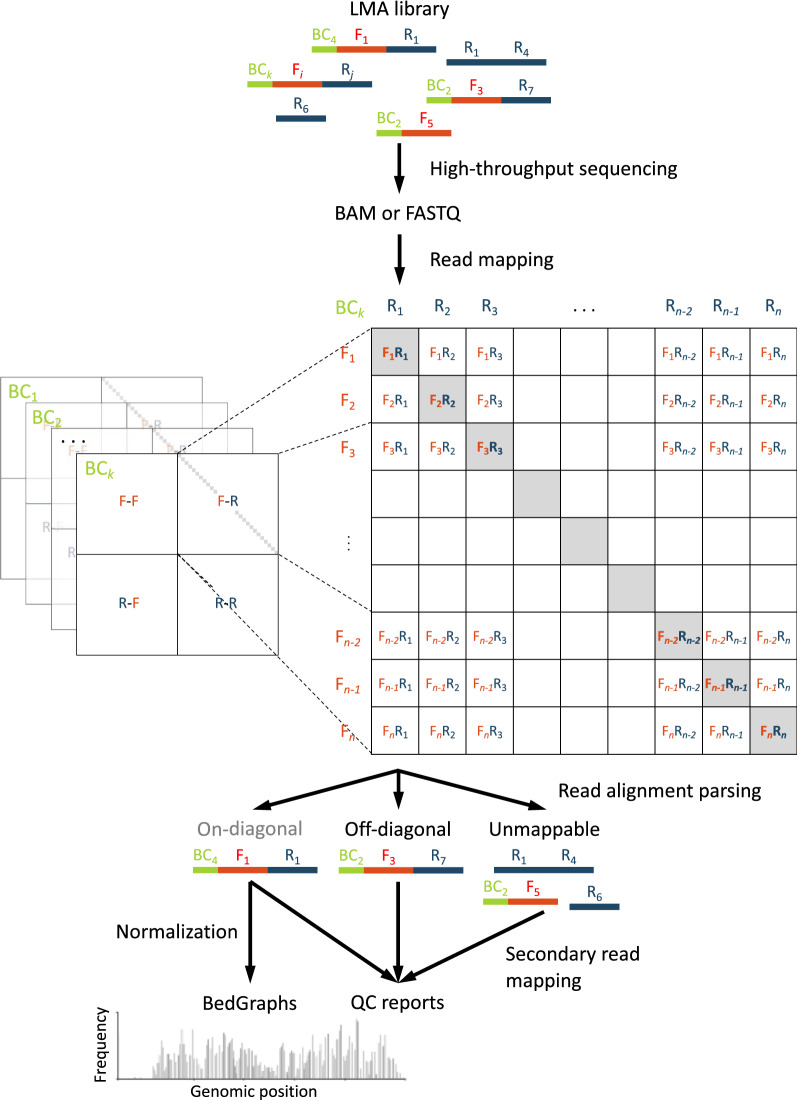
Schematic of the LAMPS analysis pipeline. LMA reads are mapped to the set of possible primer pairs (F-F, F-R, R-F, and R-R). If the sequenced library is multiplexed, the frequency count of each primer pair per barcode is obtained. Reads that are too short or could not be mapped initially (‘Unmappable’) are remapped to individual primer sequences. For 1D data, ‘On-diagonal’ read counts of expected primer pairs (gray entries of the F-R quadrant) are then normalized and outputted in bedGraph format. For 2D data, the entire F-R quadrant is provided as output in raw contact frequency matrix format

## Main text

### Materials and methods

Here we describe the Ligation-mediated Amplified, Multiplexed Primer-pair Sequence (LAMPS) analysis pipeline. LAMPS is a computational tool for mapping and analyzing sequence-specific LMA reads.

#### Input

LAMPS takes as input a FAST-Quality (FASTQ) or Binary Alignment Map (BAM) file(s) obtained from the sequencing of a (possibly multiplexed) LMA-based library, together with a text file containing primer sequences and a configuration file describing optional normalization coefficients and barcode sequences.

#### Mapping

LAMPS first uses either Bowtie 2 [8] (recommended) or Basic Local Alignment Search Tool (BLAST) [9] to map reads to the expected products containing all possible concatenations $b \cdot p_{1} \cdot p_{2}$, where *b* is a BarCode (BC), and $p_{1}$ and $p_{2}$ each are either a F or R primer. Primer-pair counts are then tabulated for each BC (see matrix representation in Fig. 1). Reads that are either too short to map as a ligation product (i.e., less than the number of nucleotides to the primer-ligation junction of the shortest primer pair) or that do not map to the database (both cases termed ‘Unmappable’) are re-mapped to individual barcode BC-F (if needed), F, and R primer sequences for Quality Control (QC) reporting. QC reports are provided at both stages of mapping to identify underperforming primers and potential errors occurring within the protocol (i.e., human error, PCR artifacts, sequencing errors, etc.).

#### Normalization

For each barcode, primer-pair read counts are normalized to Reads Per Million (RPM). When applicable (e.g., for 2C-ChIP libraries), tracks are normalized by input DNA counts and optionally corrected for sample-specific DNA density. Density correction is based on TaqMan quantification of total DNA yield following immunoprecipitation, as well as various dilution steps that occur in the preparation of pooled libraries as detailed in Wang and Cameron et al. [3].

#### Output

LAMPS’s outputs depend on whether the experiment produces one- (1D) or two-Dimensional (2D) data (2C-ChIP and 5C, respectively). In the former case, raw and normalized primer-pair read counts are provided in bedGraph format for easy integration with most genomic browsers. For 2D data, raw interaction matrices at native resolution (e.g., that of individual restriction fragments for 5C) are provided.

Included within LAMPS’s output are QC plots and reports to characterize the processed library. Library characterization includes, but is not restricted to, primer pair efficiency, raw and normalized read count comparison, and heatmaps describing the read count distribution.

### Implementation

LAMPS is a Unix-based (Linux and MacOS) command line pipeline, available in either Python v2 or v3 (versions 2.7.15 and 3.8.1 tested, respectively). Source code is available at: https://github.com/BlanchetteLab/LAMPS

The only dependencies of LAMPS are local installations of either the Bowtie 2 (recommended) or BLAST read aligner and (optional) Sequence Alignment Map tools (SAMtools) [10] (versions 2.3.4.2, 2.5.0+ and 1.3.1 tested, respectively). Included within the LAMPS GitHub repository are example 2C-ChIP and 5C datasets.

### Conclusion

LAMPS is a simple and easy-to-use computational tool for analyzing (multiplexed) sequence-specific LMA data. To the best of our knowledge, LAMPS is the first computational pipeline to provide thorough QC reporting of LMA primers. This reporting enables easy identification of problematic primer pairs during the design and data analysis of LMA experiments. To ensure LAMPS’s ease of use, the pipeline natively handles multiplexed libraries that may result from a typical LMA protocol. In addition, the standardized format of LAMPS output allows data to be easily integrated with downstream analysis pipelines and quickly studied in the context of other genomic tracks.

## Limitations

LAMPS only corrects for known biases of 2C-ChIP data. Unknown primer biases that result from the 2C-ChIP protocol may contribute to erroneous results. LAMPS also does not perform fragment-bias normalization for 5C libraries and is expected to be run upstream of 5C bias-normalization pipelines. Finally, LAMPS is not currently available on Microsoft Windows. These limitations may be addressed in future iterations of the LAMPS software.

## Data Availability

LAMPS software and example input for 2C-ChIP and 5C libraries are made publicly available at https://github.com/BlanchetteLab/LAMPS and 10.5281/zenodo.3858109 (GitHub repository and Zenodo archive, respectively).

## Abbreviations

BAM: Binary Alignment Map
BC: BarCode
BLAST: Basic Local Alignment Search Tool
BWA-SW: Burrows-Wheeler Aligner’s Smith-Waterman
DNA: DeoxyriboNucleic Acid
F: Forward primer
FASTQ: FAST-quality
LAMPS: Ligation-mediated Amplified, Multiplexed Primer-pair Sequence analysis pipeline
LMA: Ligation-Mediated Amplification
PCR: Polymerase Chain Reaction
QC: Quality control
QIIME: Quantitative Insights Into Microbial Ecology
R: Reverse primer
RPM: Reads Per Million
SAM: Sequence Alignment Map
TMAP: Torrent Mapping Alignment Program
1D: One-Dimensional
2C-ChIP: Carbon Copy-Chromatin Immunoprecipitation
2D: Two-Dimensional
5C: Chromosome Conformation Capture Carbon Copy
